# Rhodophyta as Potential Sources of Photoprotectants, Antiphotoaging Compounds, and Hydrogels for Cosmeceutical Application

**DOI:** 10.3390/molecules27227788

**Published:** 2022-11-12

**Authors:** Noer Kasanah, Maria Ulfah, Okmalisda Imania, Annisa Nur Hanifah, Muhammad Idham Darussalam Marjan

**Affiliations:** 1 Department of Fisheries, Faculty of Agriculture, Universitas Gadjah Mada, Yogyakarta 55281, Indonesia; 2 Integrated Agrocomplex Laboratory, Faculty of Agriculture, Universitas Gadjah Mada, Yogyakarta 55281, Indonesia; 3 Department of Chemistry, Faculty of Mathematics and Natural Sciences, Universitas Gadjah Mada, Yogyakarta 55281, Indonesia

**Keywords:** red seaweed, photoprotective, mycosporine like amino acid, hydrogel

## Abstract

Seaweeds are macroscopic, multicellular, eukaryotic and photosynthetic organisms, and are a source of chemical diversity with powerful biological activities for diversified industrial applications including cosmeceuticals. Red seaweeds (Rhodophyta) are good sources of Mycosporine-like amino acids (MAA) for photoprotectant and antiphotoaging compounds. In addition, Rhodophyta are also good sources for hydrogel compounds that are used widely in the food, pharmaceutical and cosmeceutical industries as gelling agents, moisturizers or for their antiphotoaging effects. Our survey and ongoing studies revealed that the biodiversity of Indonesian Rhodophyta is rich and is a treasure trove for cosmeceutical agents including MAA and hydrogels. This study delivers valuable information for identifying potential red seaweeds in screening and searching for cosmeceutical agents.

## 1. Introduction

Cosmetic products are an essential element in human society and have been used since civilization began. The Food and Drug Administration (FDA) defines a cosmetic as a product (excluding pure soap) intended to be applied to the human body for cleansing, beautifying, promoting attractiveness or altering the appearance [1]. European Commission (EC) regulation No 1223/2009 defines cosmetics as any substance or mixture intended to be placed in contact with the external parts of the human body (epidermis, hair, nails, lips and external genital organs) or with the teeth and the mucous membranes of the oral cavity with the exclusive or principal objective to clean, perfume or protect them, change their appearance or keep them in good condition. The term “cosmeceuticals” is not recognized by FDA the [1] but is used by the cosmetics industry and consumers to represent the cosmetic products that have medicinal or drug-like properties. The term contains words “cosmetic” and “pharmaceutical” and implies that the cosmetics product contains active ingredients or substances with therapeutic effects for treatment [2,3,4]. The active substances for skin benefit, such as phytochemicals, essential oils, vitamins, antioxidants, photoprotectants, etc., act on the skin to improve the skin’s health by reducing or treating skin problems such as wrinkles, dark spots, hyperpigmentation, skin aging and acne [5]. In the modern world, cosmetics are a part of one’s daily routine and personal care. The cosmetic industry is a competitive global trade with a market size valued at USD 341.1 billion in 2020 and is expected to be worth USD 480.4 billion by 2030 with a compound annual growth rate (CAGR) of 5.1% from 2021 to 2030 [6]. The global skincare products market size was valued at USD 130.50 billion in 2021 and is expected to expand at a compound annual growth rate (CAGR) of 4.6% from 2022 to 2030. Escalating demand for face creams, sunscreens and body lotions across the globe is expected to have a positive impact on the market growth over the forecast period [7].

In recent decades, the “back to nature” transformation of lifestyle has risen the interest in natural products for health, green environment and sustainability. As a result, consumers demand products from natural resources, safe, effective, healthy and eco-friendly processes [8]. The commitment of the cosmetics industry in the discovery of new natural bioactive compounds is triggered by market demand and the need to replace existing compounds. The targets are effective, non-allergenic, safe, inexpensive and made from sustainable natural compounds to deliver innovative products and solutions that meet consumers’ expectations [4,9,10]. Natural product-based cosmeceuticals are derived from a variety of sources such as plants, herbs, vegetables, legumes, oils, animals and marine biota. They are harvested from conventional agricultural production, mariculture mainly on fields or in greenhouses, biotechnological methods such as tissue cultures, hydroponic systems, fermentation of genetically modified organisms and microalgae cultures or re-use of food and agricultural waste [4,8,11]. 

Oceans cover over 70% of the Earth, being home to up to 90% of the organisms on the planet. Marine biotechnology (blue biotechnology) refers to the utilization of marine resources and biotechnology tools for various applications in industrial, human health, or environmental industries. Marine ecosystems harbor a largely unexplored biodiversity, which offers considerable potential for blue biotechnology and blue economy including cosmeceuticals [12]. The oceans supply material, new extracts and molecules for active ingredients, excipient or additive, for cosmeceutical formulas that meet the expectations of consumers attracted by natural products [13,14]. Marine organisms that are used in the cosmetics industry are sponges, seaweeds, shark fish, marine turtles, coral, jelly fish and hydras. They produce wide array molecules such as polysaccharides, fatty acids (sophorolipids, rhamnolipids and mannosylerythritol) and proteins that are widely used on the skin. Blue cosmeceuticals consist of a large number of natural compounds isolated from various marine resources [15,16,17]. Seaweeds offer excellent potential contributions for cosmeceuticals due to their valuable substances, sustainability, low cytotoxicity and low allergens [2,5,18]. 

Seaweeds are macroscopic alga (macroalgae), aquatic, photosynthetic organisms, which lack true roots, leaves and stems. Seaweeds are a primary producer and play a noteworthy role in the marine tropic chain and bioresources of the oceans. They have ecological functions in the marine ecosystem by supplying oxygen to the sea, providing shelter for marine microorganisms and fishes breeding. Their secondary metabolites have effects on intra- and inter-populational interactions for structuring the seaweed population. Some seaweeds absorb heavy metals and pollutants and thus can be applied for the bioremediation to remove pollutants from the water [19,20,21]. Seaweeds have the capacity to survive in the environmental stresses that they are exposed to. They produce organic osmolytes during stress conditions, which also act as antioxidants and heat protectants. Most seaweeds possess antioxidant activities due to their ability to live in stress conditions. Under UV exposure in the intertidal zone, seaweeds, mainly red seaweeds, synthesize UV-absorbing compounds to protect against UV radiation. Thus, red seaweeds are more resistant against UV than terrestrial plants [22,23]. Seaweeds have developed complex and unique metabolite pathways to produce primary and secondary metabolites. Proteins, amino acids, polysaccharides and fatty acids are primary metabolites involved in physiological functions under normal growth conditions. Alkaloid, terpenoid, polyphenolic and halogenated compounds are secondary metabolites which are synthesized by seaweeds during environmental pressure due to exposure to UV radiation, changes in temperature, salinity pollutants or pathogens [10]. 

**Figure 1:**
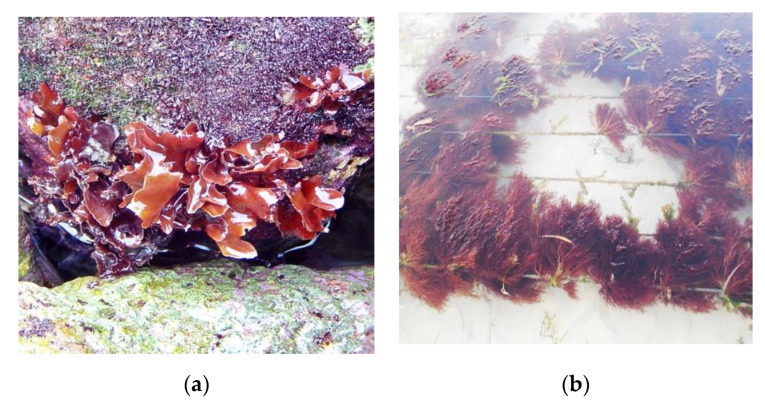
Red seaweeds grow on (**a**) rocky substrate and (**b**) float in water.

Most seaweeds live almost exclusively in the shallow coastal waters at intertidal zones. They live as epiphytes or attach to substrates such as rocks, stones, pebbles, dead corals or other substrates (Figure 1). Seaweeds are found floating and submerged in the intertidal zone of ocean tropical waters to deep sea [10,24]. In the history of humans, seaweeds have been utilized for food, polysaccharide, biofertilizers, cosmetics, papermaking and, recently, as biobased fuels [25,26].

World aquaculture production is dominated by five genera: Saccharina, Undaria, Porphyra, Eucheuma/Kappaphycus and Gracilaria. The species *Kappaphycus alvarezii* and *Eucheuma* spp., *Saccharina japonica*, *Gracilaria* sp., *Undaria pinnatifida*, *Porphyra* (Pyropia) sp. and *Sargassum fusiforme* represent 98% of the world’s seaweed aquaculture [27,28]. Approximately 150 seaweed species are consumed as human food and about 250 seaweed species are commercially exploited worldwide [29,30]. Seaweeds have demonstrated benefits for human skin health, such as anti-melanogenesis, antiaging, photoprotection, antiwrinkle, moisturizer, anti-inflammatory, anticancer and antioxidant properties, as well as certain antimicrobial activities, such as antibacterial, antifungal and antiviral activities [4,31,32,33]. Molecules from seaweeds have been applied as a cosmetic active ingredient such as mycosporine-like amino acids, pigments and phenolic compounds, while primary metabolites such as agar, alginate and carrageenan are used for the products’ consistency [18]. Hydrocolloids from seaweeds are used as moisturizing and thickening agents for the formulation of cosmetics [34]. 

The best way to describe the potentialities of seaweed for the cosmetic industry is to start determining valuable bioactive compounds for cosmeceuticals. This review is designed to deliver an overview of the application of red seaweeds for cosmeceuticals by highlighting the main compounds responsible for photoprotective and antiphotoaging products and hydrogels. In addition, the potential and prospects of developing Indonesian red seaweeds for cosmeceuticals are also reviewed. The reference sources were searched by referring to Scopus, Science Direct and Google Scholar using keywords such as red seaweed, red seaweed cosmeceutical, Mycosporine-like amino acids, red seaweed gel, red seaweed polysaccharide, photoprotectant and antiphotoaging. We collected references from 2010 to 2022 from all regions in the world. 

## 2. Red Seaweeds (Rhodophyta)

**Figure 2:**
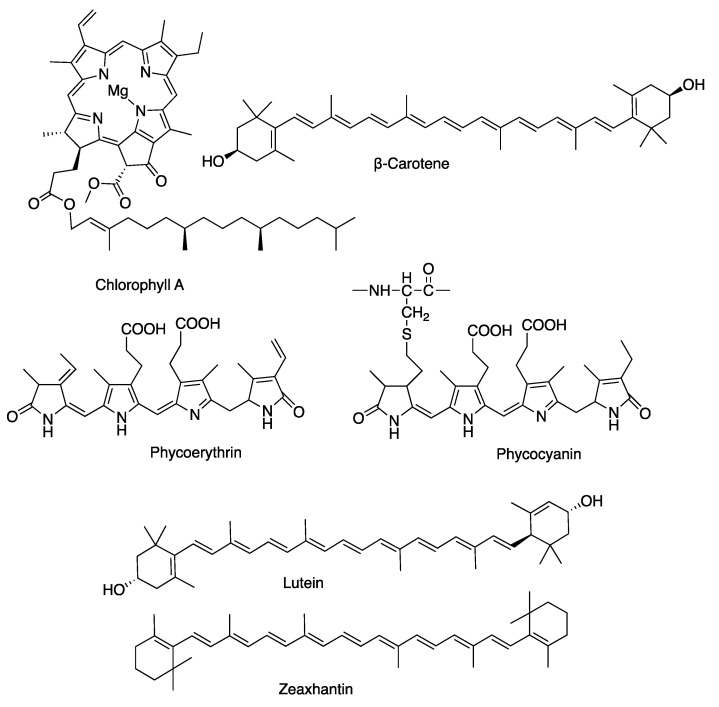
Pigments of Rhodophyta.

Seaweeds are classified based on their pigment composition: green (Chlorophyta), brown (Ocrophyta, Phaeophyceae) and red (Rhodophyta). Chlorophyta contain chlorophyll *a* and *b*, while Phaeophyta are rich in fucoxanthin and xanthophyll. Rhodophyta contain photosynthetic pigments, such as chlorophyll a, phycobilins, such as R-phycocyanin and R-phycoerythrin, and carotenoids such as β carotene, lutein, zeaxanthin (Figure 2) [35,36,37]. The biodiversity was counted as 1800 species of Phaeophyta, 6200 species of Rhodophyta and 1800 species of Chlorophyta [38].

**Figure 3:**
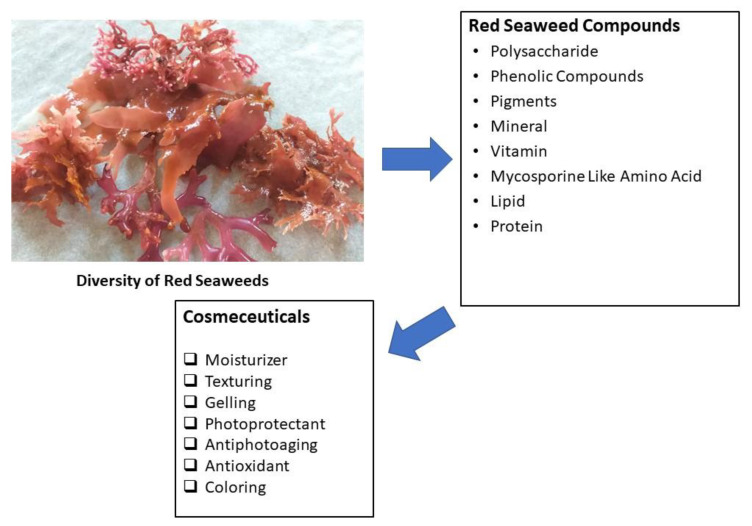
The diversity of red seaweeds and potential applications for cosmeceuticals.

The red seaweeds (Phylum Rhodophyta) Class Rhodophyceae is a very large group of species that are predominant in marine environments. The biodiversity of red seaweeds is higher than green and brown seaweeds. There are 6000 different species of Rhodophyta that live in the freshwater and marine environment. About 98% of the red seaweed species are marine organisms. They can be found in the intertidal zone, coral reef, tide pools and marine tropical areas. Most red seaweeds play vital roles in the food chain and produce about 40 to 60% of the total global oxygen. The pigment phycoerythrin facilitates some red seaweeds to be able to live in the deep ocean by absorbing blue light waves [39,40]. Our field studies on intertidal zones showed that red seaweeds can be found on rocky beaches. Common species that live in the coastal areas of Indonesia are *Gracilaria* spp., *Laurencia* spp., *Gelidium* spp., *Acanthophora* spp. and *Palmaria* spp. (Figure 3).

Red seaweeds have economic value and are worth billions of dollars every year. At least 125 different species of red seaweeds are used worldwide with different applications. It is estimated that, of the 125 species of red seaweed with economic applications, 79 are used as food for human consumption, 33 in agar production and 27 in carrageenan production [39]. Red seaweeds are the major sources of hydrocolloid agar and carrageenan for industrial purposes [41]. Other potential molecules are essential fatty acids, phycobiliproteins, vitamins, minerals and diverse types of secondary metabolites. Red seaweeds have been used as a source of food for thousands of years as they are high in vitamins and minerals and are a rich source of calcium, magnesium, carbohydrates and antioxidants. They are sources of dietary fiber as they have the ability to promote healthy circulation, lower bad cholesterol and regulate blood sugar levels [3,30,42]. They are also involved in nourishing skin, boosting the immune system and contributing to bone health [40]. Secondary metabolites of Rhodophyta are abundant and high in chemical diversity, often used for different applications such as photoprotectant and antiphotoaging products [43]. 

## 3. Photoprotectant and Antiphotoaging Properties

UV radiation causes harmful effects to human skin such as erythema, edema, hyperpigmentation, photoaging and skin cancer. Thus, the need to protect human skin has increased the demand of sun protection products that contain UV-absorbing molecules. Natural products have gained considerable attention for use in sunscreen products and have confirmed the trend of natural cosmetics. Seaweeds produce photoprotective substances such as sulfated polysaccharides, carotenoids, polyphenolic compounds and mycosporine-like amino acids [13,16,44]. This review focuses on mycosporine-like amino acids. Red seaweed is one of the natural resources containing an abundance of types of mycosporine-like amino acids, which have shown potential of photoprotection, antiphotoaging and antioxidant properties for safeguarding against the damaging effects of UV radiation exposure. 

### 3.1. Photoprotectant

The ground levels of ultraviolet radiation (UVR) are increasing gradually due to the high pollutant level and reduction of cloud cover in the stratospheric ozone layer [45]. Marine organisms that are exposed directly to the sunlight have evolved multiple photoprotective mechanisms to defend against the high level of UVR such as producing antioxidant compounds, repairing DNA and releasing UV-absorbing compounds [46,47,48]. High exposure of UVR creates the production of reactive oxygen species (ROS), oxidative stress and DNA damage. Marine organisms produce antioxidant compounds by catching free radicals to respond to the ROS [49] and accumulate photoprotective compounds such as mycosporine-like amino acids (MAAs) to absorb UVR [50]. Diverse aquatic organisms including seaweed, cyanobacteria, phytoplankton and marine animals produce MAAs. The photoprotective function of MAAs in marine organisms can be inferred from their efficiency in absorbing ultraviolet-A (320–400 nm) and ultraviolet-B (280–320 nm) radiation due to their high molar absorption coefficients and frequent observations correlating higher MAAs’ concentrations with higher levels of UVR [51,52,53,54].

Mycosporine-like amino acids are group of secondary metabolites with a core structure of a cychlohexenone ring or cyclohexenimine ring. The compounds are of small molecular weight (less than 400 Da), water soluble, colorless, uncharged and are a strong UVR absorber. The UV absorption maximum spectrum of MAAs appears between 310 and 360 nm depending their molecular structure [55]. MAAs also have high molecular absorptivity (ε = 12.400–58.800 M^−1^·cm^−1^). MAAs are the strongest UVA-absorbing compounds in nature, and they are also effective against UVB radiations, which explains their potential role in photoprotection. Unique characteristics and bioactivities that have been attributed to MAAs make them good candidates for cosmeceutical applications such as photoprotectant agents [56]. These molecules are excellent UV-absorbing compounds with low toxicity, especially high stability and good antioxidant activity [57,58]. Due to its high water solubility, MAAs are predominantly present and dispersed in the cytoplasm and can be easily extracted using polar solvents ethanol, methanol or in combination with water [59].

The structures of MAAs are varied and classified based on the amino acids substituents and the ring system. The different amino acid substituents into the core structures are the key to the diversity of MAAs. The first group is a cychlohexenone ring with a single modified amino acid and the second is a cyclohexenimine ring which has two amino acid substituents [52]. Organisms’ biosynthesis of MAA is through the shikimate pathway or pentose phosphate pathway. In the shikimate pathway, the parent molecule 3-deoxy-D-arabinoheptulosinate 7-phosphate (DAHP) produced 3-dehydroquinate (3-DHQ) through 3-dehydroquinate synthase (DHQS) and it is transformed into gadusol and then into 4-deoxygadusol (4-DG). In the pentose phosphate pathway, the sedoheptulose 7-phosphate (SH 7P) yields 4-deoxyadiosol (4-DG) [60]. Harsh environment and UV light induce the biosynthesis of a wide array of MAAs as a natural sunscreen and antioxidant. 

**Figure 4:**
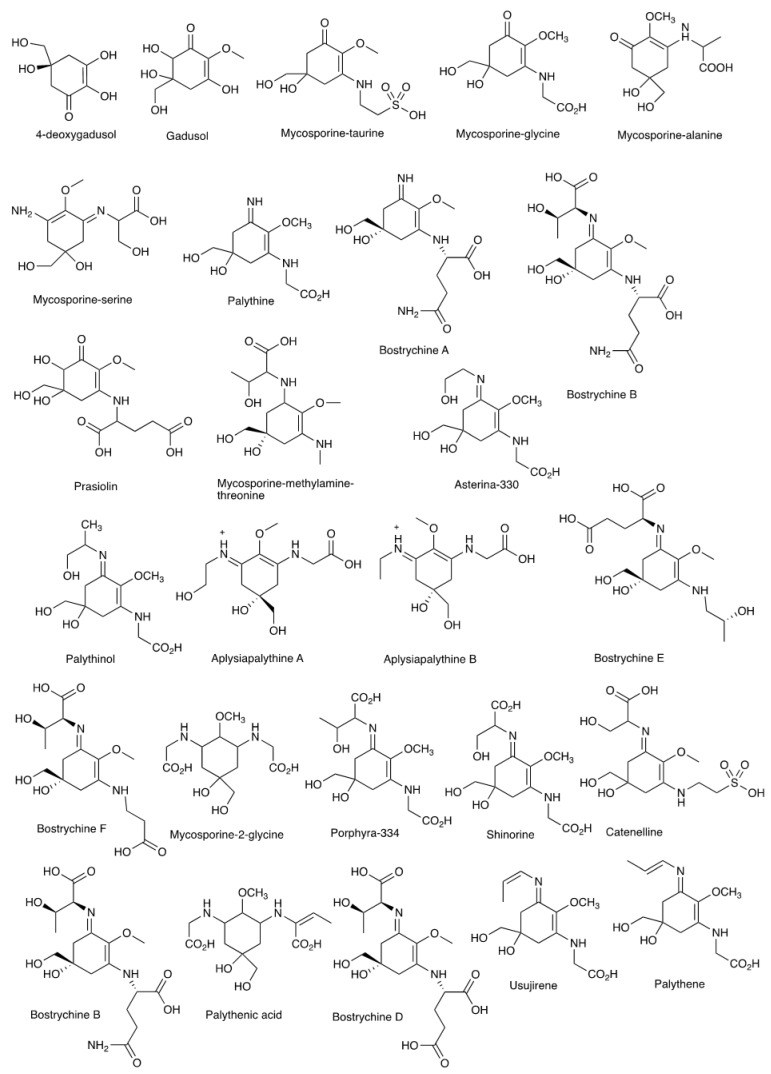
Structure of MAAs from red seaweed.

Red seaweeds are considered the most productive and rich sources for MAAs; thus, they are considered as promising sources of MAAs for the cosmeceutical industry, especially by their photoprotection capacity. A comprehensive study by [55] concluded that there are 572 species of seaweeds that produce MAA, primarily found in Rhodophyta (486 species), Chlorophyta (45 species) and Phaeophyta (41 species). The content of MAAs is varied in different species of red seaweeds. Genus Gracilaria is the source of different types of MAAs. *Gracillaria birdiae* and *G. domingensis* produce shinorine, palythine and porphyra 334, while *G. tenuispitata* produces the same compounds with the addition of asterina 330 and palythinol [61]. *Gracilaria confervoides* as well as *Gelidium amansii* were reported to contain gadusol, shinorine palythine porphyra-334, palythenic acid and three unknown MAAs detected at 328, 330 and 331 nm. Analysis of *Bangia fuscopurpurea* yielded shironine, palythine, porphyra-334 and palythenic acid [62]. *Palmaria palmata* or Dulse is an edible seaweed and source of shinorine, palythine, asterina-330, porphyra-334, usujirene and palythene based on analysis by employing shinorine, palythine, asterina-330, porphyra-334, usujirene and palythene [63]. Determination of MAAs from 23 species of red seaweeds of Australia found that shinorine, palythine, asterina-330 and porphyra-334 are the most common MAAs in all species. In addition, there are a few red seaweeds that produce aplysiapalythine A, mycosporine-glycine, mycosporine-alanine-glycine, aplysiapalythine B, mycosporine-methylamine-threonine, usujirene and palythine [52]. Other studies on red seaweeds have identified different types of MAAs such as palythene, catenelline, mycosporine-2-glycine, palythenic acid, palythinol, prasiolin and bostrychines A–F. Some seaweeds produce specific types of MAAs such as aplysiapalythine A, aplysiapalythine B (*Gigartina macrocarpa, Pyropia columbina, Porphyra umbilicalis*), catenelline (*Catenella repens*) and bostrychines A–F (*Bostrychia calliptera*) [50,62,64,65]. The list and structure of MAAs from red seaweeds are presented in Table 1 and Figure 4.

The chemical diversity and the accumulation of MAAs in seaweeds depend on different parameters such as water depth, harvest time, nutrient availability, pH, temperature and irradiation [66,67]. The exposure to solar radiation is correlated to the highest concentration of MAAs. Intertidal seaweed at low latitudes contains high MAAs concentration rather than species inhabiting the subtidal zone and a high latitude. Intertidal seaweeds are exposed to a variety of abiotic and biotic factors which cause chemical diversity not only between individuals but also within individuals [68]. Extensive studies on the content of MAAs in red seaweeds have classified three groups of red seaweed-producing MAAs. The first group, including 65 species, belong to order Balliales, Ceramiales, Corallinales, Nemaliales and Rhodymeniales, exhibited low total MAAs contents (<1 mg/g DW). A second group with a higher concentration of total MAAs (1~2 mg/g DW) consists of 52 species. The last group, including 216 species and the orders Bangiales, Gelidiales, Gigartinales and Gracilariales macroalgae, showed the highest total MAAs (>2 mg/g DW) [55]. Several research studies showed the variation of the level of MAAs in species of red seaweeds. The endemic red seaweed of New Zealand *Pyropia plicata* produced porphyra-334 and shinorine with the total amount varying between 8.4 and 13.7 mg/g DW [69]. *Chondracanthus chamissoi* (Gigartinales) and *Gelidium lingulatum* (Gelidiales) from the southeast Pacific coast yielded shinorine and palythine in both species ranging from 0.8 to 6.8 mg/g [70]. *Porphyra umbilicalis* is a potential producer of MAAs which has been used in commercial sunscreen products Helioguard 395 and Helinori due to photoprotective and antiaging activities. The concentrations of MAAs of *Porphyra umbilicalis* (5.2 ± 0.40 mg/g DW) consist of myc-glutamine, palythine, palythinol, asterina-330, shinorine and porphyra-334 [71]. *Porphyra umbilicalis* from the southern Iberian Peninsula produced >10 mg/g DW of MAAs (asterina-330, palythine, palythinol, shinorine and porphyra-334). Asterina-330 and porphyra-334 represented 72% and 23% of the total MAAs, respectively [72]. Six types of MAAs (palythine, asterina-330, porphyra-334, mycosporine-glycine, mycosporine-alanine-glycine and aplysiapalythine B) were detected in *P. umbilicalis* collected from Heligoland, Germany. The concentration of mycosporine-glycine was reported as 10.21 ± 0.24 mg/g DW [73]. The methods and solvent systems for extracting MAAs from red seaweeds contribute to the yields of MAAs [62]. 

The chemical diversity of MAAs in red seaweeds differs in one species in response to geographic regions. The investigation of *Bostrychia calliptera* revealed a different phytochemical profile corresponding to three categorized lineages. The first lineage was found in North and South America and Australia. Lineage 2 was found in Australia and Southeast Asia and lineage 3 was encountered in Central and South America. The contents of MAAs in samples of each lineage are presented in Table 2. Therefore, the type of MAAs could be varied within the species in different spatial distributions [74].

Seasons have contributed to the level of accumulation of MAAs in wild seaweeds. Three species of sub-Antarctic seaweeds, *Nothogenia fastigiate*, *Iridaea tuberculosa* and *Corallina officinalis*, exhibited the highest MAA content in different seasons of the year. The highest content of MAAs (>1 mg/g) was detected in *N. fastigiata* collected during spring and *I. tuberculosa* during winter. The calcareous *C. officinalis* showed the lowest total of MAAs not exceeding 0.4 mg/g during summer. Porphyra-334 was the main component in *N. fastigiata*, whereas *I. tuberculosa* and *C. officinalis* exhibited a high content of palythine [75]. The low amount of MAAs in *C. officinalis* was associated with the cell wall containing calcium carbonate, which effectively reduces the absorption level of UV radiation [76]. A survey of three red intertidal seaweeds from New Zealand showed that *Bostrychia arbuscula* and *Schizymenia* spp. produced higher MAA concentrations in summer, while *Champia novae-zelandiae* displayed the lowest level of MAAs in summer [77]. The different seasonal dynamics in MAAs’ accumulation is not specifically related to the seasonality of solar radiation but to the variation of environmental conditions at local levels. The results occurring in natural conditions revealed a high seasonal variability that can be related to a combination of environmental parameters, including light, nutrients, temperature, salinity and pH, which may have synergistic or antagonistic effects. The total MAAs content was significantly influenced by the increase in light and irradiance. A study reported the changes in MAAs composition of the red edible seaweed *Palmaria palmata* with the higher MAAs content occurring in April and May, due to the light increase [78]. Increasing light and irradiance also directly affected MAAs content in *Palmaria palmata*, *Chondrus crispus* and *Palmaria dioica* during the spring season [51].

The nutrient availability could have a significant influence on MAAs since their synthesis seemed to be limited in summer, despite significant exposure to light. A positive relationship between N availability and accumulation of photoprotective compounds, such as mycosporine-like amino acids (MAAs), has been reported in different species of red seaweed. MAAs content in *Gracilaria tenuistipitata* increased up to eightfold with the gradual addition of NO_3_^–^ to a concentration of 0.5 mM [79]. *Agarophyton vermicullophylum* from Portugal produced high MAAs content in spring, due to the nitrogen (N)-enriched waters [80]. The study of carrageenan-producing red seaweed *Mazzaella laminarioides* revealed that the highest MAAs content is available with the supply of NO_3_^–^. Four MAAs were identified from *M. laminaroides*: asterina-330, shinorine, palythine and mycosporine-glycine, while the accumulation of shinorine and palythine varied according to NO_3_^–^ supply [81].

MAAs possess multiple roles to aid against environmental stress conditions such as osmotic regulation, desiccation, control reproduction, nitrogen reservoir, thermal and salt stresses and toxicity by heavy metals [22,23,82,83]. MAAs from red seaweeds are reported as versatile metabolites with multiple functions including antioxidant, anti-inflammatory, antiaging, anticancer, and wound healing properties [63,84,85,86]. Investigation of porphyra-334 isolated from *Porphyra yezoensis* exhibited a protective effect on human skin fibroblasts against exposure to UV-A radiation by increasing cell viability up to 88%. Based on the dose-dependent manner similar to 40 µM, porphyra-334 also inhibited the intracellular accumulation of ROS in human skin fibroblasts damaged by UVA-induced oxidant stress [87]. Orfanoudaki et al. [88] provided evidence of the ability of shinorine, porphyra-334, mycosporine-glycine-alanine and bostrychine B from *Bostrychia scorpioides* to stimulate human keratinocyte migration *in vitro*. All tested MAAs in two concentrations of 1 µM and 10 µM showed significant migration and narrowing of the scratch area after 24 h (*p* < 0.05). The interaction between MAAs with human keratinocytes indicates wound closure ability. Palythine isolated from *Chrondus yendoi* provided protection from UVR-induced ROS production and exhibited antioxidant properties. Palythine showed significant activity (*p* = 0.004) compared with established antioxidant Trolox (29.1%) and asorbic acid (34.8%). Palythine also significantly reduced the SSR-irradiated (20 J cm^−2^) production of oxidizing species in Ha-CaT immortal human keratinocytes [89]. 

**Figure 5:**
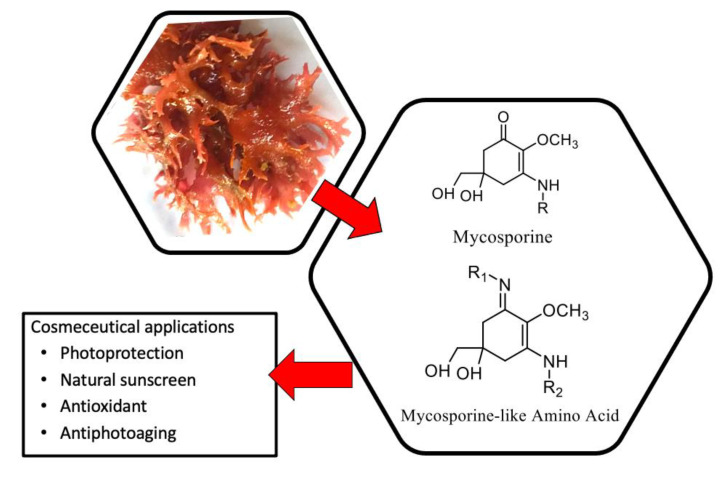
Mycosporines and the applications for cosmeceuticals.

Mycosporine-like amino acids are a large family of natural UV-absorbing compounds. The significant diversity of MAAs has attracted attention since they can provide a wide range of advantages in cosmeceutical applications as photoprotectants, antiphotoaging compounds, cell proliferation activators, anti-inflammatory or anticancer agents and skin cell renewal stimulators (Figure 5). Interest in MAAs for cosmeceutical applications has been growing in the past decades [83]. Moreover, the pharmacological activities such as antibacterial, anticancer, antiviral and anti-allergic properties are promising for application in the biomedical field [90]. 

### 3.2. Antiphotoaging 

Skin aging is a natural biological process that causes wrinkles, dark spots, a decrease in skin elasticity, skin dullness and skin roughness [91]. Ultraviolet radiance is an extrinsic factor that induces a high production of reactive oxygen species (ROS) that can promote premature skin aging [92]. Ultraviolet radiation decreases skin elasticity due to collagen degradation [93]. Recent research has concentrated on marine-derived natural products as a promising source of natural bioactive molecules and some UV-absorbing compounds. Seaweed compounds are being discovered as potential new natural sunscreens, photoprotectants and antiphotoaging agents [94,95]. The use of photoprotective agents is primarily responsible for postponing the effects of photoaging by reducing the negative effects of free radicals [94]. The various categories of natural antiaging ingredients include moisturizing agents, antioxidants, sunblock ingredients, vitamins, hydroxy acids and skin-lightening agents [8].

The rising of interest in the usage of marine components over the last decades can be observed in the preliminary analysis of the presence of these ingredients in all 293 antiaging cosmetics under study, representing a total of 40 brands. Extracts from red seaweeds *Kappaphycus alvarezii* and *Chondrus crispus* are the “top 3” marine components. *Chondrus crispus* has been used since 2011, and *Kappaphycus alvarezii* application has increased in use since 2018 [95]. *Kappaphycus alvarezii* and *Acanthophora spicifera* extracts used in textile face masks for antiaging applications were reported in the cosmetic sector [96]. 

Red seaweed’s compounds that have potential as sources of antiphotoaging agents are carrageenan, mycosporine-like amino acids and phenolic compounds [84,93,97,98]. Carrageenan indicated potential antiphotoaging properties that were mediated by intracellular reactive oxygen species (ROS) scavenging activity [99]. Carrageenan activities correlate with the modulations of inflammatory responses. Modulation of inflammatory responses and antioxidant activities of carrageenan play an important role in antiphotoaging activities [98]. Carrageenan is also used to moisturize the skin. Moisturization is the first step in protecting the skin from aging. It contributes to the preservation of its appearance and to the elasticity of the skin [34].

Polyphenols act as reducing agents to protect the body’s tissues against oxidative stress. The antioxidant activity of seaweed polyphenols was found to be correlated with phenolic compounds and flavonoids, indicating their roles as free radical scavengers and ferric ion-reducing agents [100,101]. Antioxidant activity can be evaluated using a 1,1-diphenyl-2-picrylhydrazyl (DPPH) scavenging assay. Some red seaweeds possess antioxidant activity. An ethyl acetate extract of *G. changii* showed a DPPH radical scavenging effect EC_50_ of 0.51 ± 0.09 mg/mL, total phenolics content (TPC) was 21.57 ± 2.58 mg/g PGE, total flavonoids content (TFC) was 200.87 ± 3.61 mg/g RE and total carotenoids content (TCC) was 7343.59 ± 148.65 µg/g BE [101]. Ethanol: _d_H_2_O (4:1) extracts of *Gracilaria corneum* and *Osmundea pinnatifida* showed DPPH assays with a result of 4 µmol/g DW [71]. Antioxidant activities were measured from an extract of *Halymenia durvillaei* and determined by ferric reducing antioxidant power (FRAP) and trolox equivalent antioxidant capacity (TEAC) assays. The results from the FRAP and TEAC assays were 182.29 ± 13.35 µM/mg dry extract and 1.67 ± 0.04 mM/mg dry extract [100]. 

The major phenolic acids such as gallic acids, protocatechuic acid, chlorogenic acid and gentisic acid that are useful as antiaging agents have been identified in the extracts of *Palmaria palmata*, *Porphyra purpurea*, *Chondrus crispus*, *Mastocarpus stellatus*, *Gracilaria vermiculophylla* and *Polysiphonia fucoides* [102]. Gallic acid reduced skin dryness and wrinkle formation by inhibiting MMP (matrix metalloproteinase)-1 secretion and regulating elastin, type I procollagen and transforming growth factor-1 [103]. Protocatechuic acid has been reported to be high in antioxidants. Protocatechuic acid has potential as an antiwrinkle and antiaging agent. Protocatechuic acid showed the ability to encourage the production of type 1 collagen in human dermal fibroblasts [104]. Chlorogenic acid is well-known for its ability to face oxidative stress. By reducing ROS levels, chlorogenic acid has the potential to be utilized as a substance that prevents aging [105]. The DPPH scavenging activities (EC_50_, µg/mL) of *Palmaria palmata*, *Porphyra purpurea*, *Chondrus crispus*, *Mastocarpus stellatus*, *Gracilaria vermiculophylla* and *Polysiphonia fucoides* extracted with ethanol were 1166.7 ± 18.0 µg/mL, 1233.3 ± 24.0 µg/mL, 1000.0 ± 25.0 µg/mL, 1366.7 ± 33.0 µg/mL, 1033.3 ± 2.6 µg/mL and 7.5 ± 1.4 µg/mL. On the other hand, EC_50_ aqueous extracts were 571.4 ± 11.4 µg/mL, 729.1 ± 80.0 µg/mL, 530.7 ± 27 µg/mL, 1500 ± 19.3 µg/mL, 166.7 ± 17.0 µg/mL and 111.4 ± 2.2 µg/mL [102]. 

**Figure 6:**
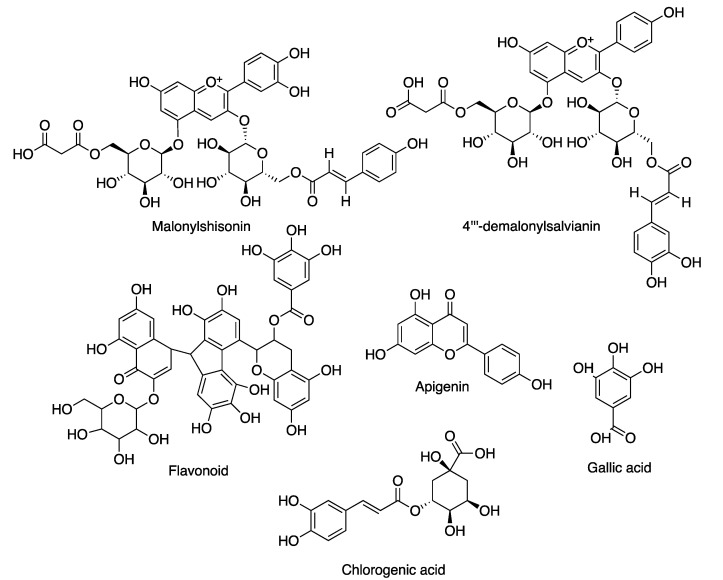
Structures of antiphotoaging compounds from red seaweeds.

Flavonoids are used in antiaging treatments because they have antioxidant and antiaging properties. Flavonoids help to increase skin hydration, skin elasticity and collagen content [106]. *Jania rubens* extract contains two such anthocyanins (malonylshisonin and 4′-demalonylsalvianin), which belong to the class of flavonoids. Anthocyanins potentially give protection from UV radiation, reduce the appearance of skin aging and inhibit inflammatory and lipid peroxidation [97]. Table 3 shows antiphotoaging agents from red seaweed and the structure of antiphotoaging compounds from red seaweed are presented in Figure 6.

**Figure 7:**
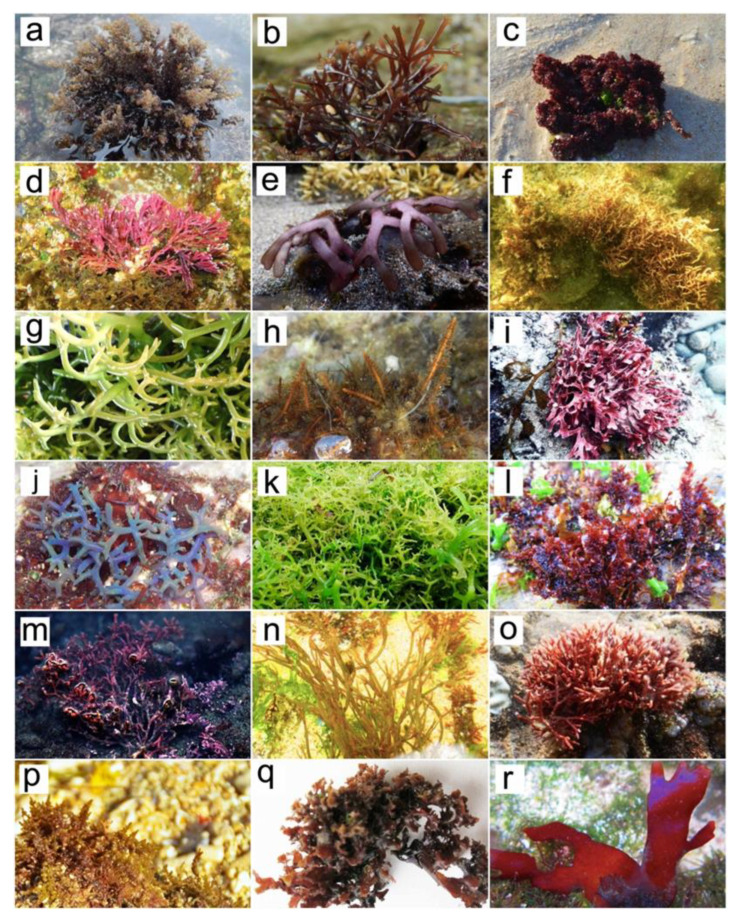
Red seaweed species from Indonesia with potential photoprotectant and antiphotoaging properties. (**a**) *Acanthophora spicifera*; (**b**) *Ahnfeltiopsis* sp.; (**c**) *Amansia* sp.; (**d**) *Amphiroa* sp.; (**e**) *Dichotomaria marginata*; (**f**) *Ganonema* sp.; (**g**) *Eucheuma cottonii*; (**h**) *Gelidiella acerosa*; (**i**) *Gymnogongrus* sp.; (**j**) *Hypnea* sp.; (**k**) *Eucheuma spinosum*; (**l**) *Gelidium* sp.; (**m**) *Galaxaura* sp.; (**n**) *Gracilaria* sp.; (**o**) *Jania rubens*; (**p**) *Laurencia* sp.; (**q**) *Mastocarpus stellatus*; (**r**) *Palmaria palmata*.

The biodiversity of seaweeds in Indonesia has not been fully explored and identified for economic value and utility in human life. In the survey on Indonesian seaweed biodiversity, we identified that red seaweeds might be potential MAAs-producing and antiphotoaging species. Red seaweeds were collected from the coastal area of Indonesia including Gunungkidul and Nusa Tenggara Timur [120,121]. The biodiversity of Indonesian red seaweeds is presented in Figure 7. The potential of Indonesian red seaweeds as sources of photoprotective and antiphotoaging compounds is compiled in Table 4.

## 4. Hydrogel

**Figure 8:**
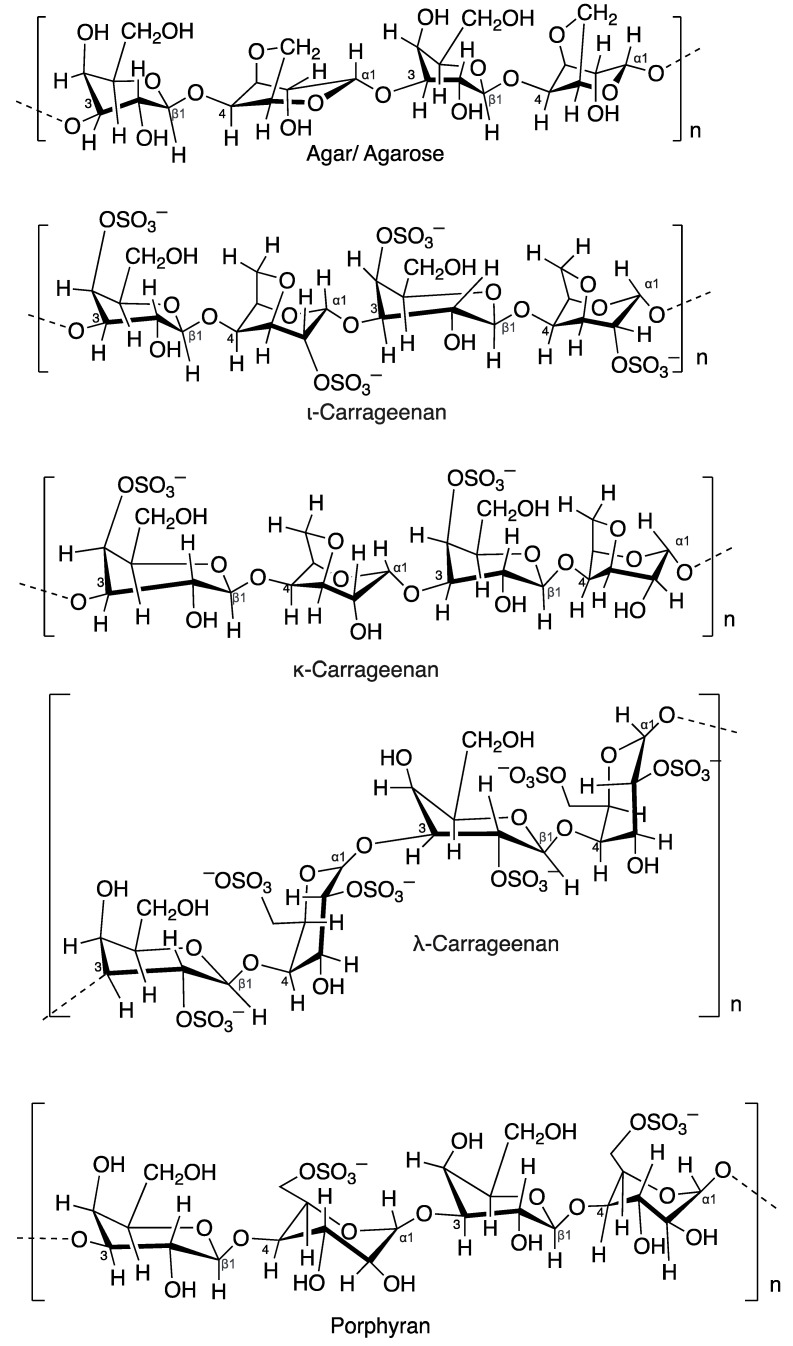
Chemical structures of agar, *iota* carrageenan, *kappa* carrageenan, *lambda* carrageenan and porphyran.

The majority of the primary metabolites found in seaweed, which count for about 60% of cell weight, are polysaccharides. Carbohydrates showed various biological activities against skin disorders including hyperpigmentation, wrinkles, dry skin disorders, skin inflammation and skin cancer [134]. The primary component of polysaccharides is a chain of monosaccharides connected by glycosidic linkages. The majority polysaccharides from red seaweeds are sulphated galactan, agar, carrageenan and porphyran with a primary structure composed mainly of galactose units with varying degrees of sulfation [3]. Red seaweeds contained types of sulfated polysaccharides, which are used in the cosmeceutical industry and act as an antiaging, anti-inflammatory, antiredness/anticouperose, antimicrobial, antioxidant, moisturizing, protective and sunscreen agents [135]. Natural sulfated polysaccharides are non-irritant and are recommended for use in anti-acne, decorative cosmetics, sun care, skincare and antiaging products. Polysaccharides from red seaweeds offer a variety of applications in the food and cosmetic industries [135]. In the present studies, sulfated polysaccharides significantly reduced intracellular ROS (reactive oxygen species) levels and they also improved the survival of UVB-irradiated cells in vitro in human skin in a dose-dependent manner [136]. These substances often function as moisturizing and antioxidant components in cosmeceuticals [32]. Table 5 and Figure 8 show polysaccharides from red seaweed that are used for cosmeceutical purposes. 

### 4.1. Agar

**Figure 9:**
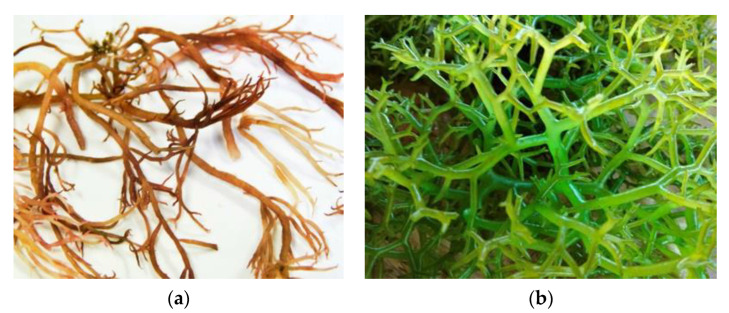
(**a**) agarophyte (*Gracilaria* sp.) and (**b**) caragenophyte (*Eucheume* spp.) from Indonesia.

Agar is a hydrophilic compound formed of agarose and agaropectin derived from many species of red seaweeds. In the pharmaceutical field, agar is used as a thickening agent and as a component of tablets or capsules that contain and release medications [5]. *Gelidium* spp. and *Gracilaria* spp. (Figure 9a.) are the most popular seaweeds used for the industrial production of food-grade agar [137]. Agar can be used to regulate the viscosity and emollience of cosmetic products. As a substitute for cocamide DEA (diethanolamine), agar from *Gracilaria* sp. has a gelling ingredient that can have a thickening effect on some products, including liquid bath soap. In the cosmetic industries, agar is frequently used as a key component in creams, as an emulsifier and stabilizer, and to regulate the moisture content in cosmetic products such as hand lotions, deodorants, foundations, exfoliants and scrubs, cleansers, shaving creams, antiaging products, facial moisturizers, liquid soaps, acne treatments, body washes and facial powders [32].

### 4.2. Carrageenan

Carrageenans are polysaccharides that are isolated from various families of the Gigartinales order (*Betaphycus gelatinum*, *Chondrus crispus*, *Eucheuma denticulatum*, *Gigartina skottsbergii*, *Kappaphycus alvarezii*, *Hypnea musciformis*, *Mastocarpus stellatus*, *Mazzaella laminaroides*, *Sarcothalia crispate*). These polygalactans are sulfated and contain a linear structure made of alternating galactose and glucose residues. From a commercial point of view, *kappa*, *iota,* and *lambda* are the three main forms of extracted carrageenan that are significant. *Iota*, *kappa*, and *lambda* carrageenans are viscosifiers and gelling carrageenans, respectively [32]. The primary source of *kappa*-carrageenan is the tropical seaweed *Kappaphycus alvarezii* or *Eucheuma cottonii* (Figure 9b), which is grown for food and is also referred to as “cottonii” in the trade. The primary species for the production of *iota*-carrageenan is *Eucheuma denticulatum* (also known as “spinosum” in trade). Under the trade name “IrishMoss”, many species of the genera Gigartina and Chondrus are used to produce *lambda*-carrageenan [138]. Indonesia produces the largest amount of carrageenan seaweed worldwide and the species *Kappaphycus* sp. and *Eucheuma* sp. have been cultured for decades [139,140]. Carrageenan plays significant roles in the cosmetic industry as a thickener, stabilizer and water-binding agent in cosmetic products. Their diverse biological activities make them suitable for use as active ingredients because of their antioxidant, photoprotective, antiaging properties, and antimelanogenic property is important for cosmeceutical agents [134,141,142]. The carrageenans (*kappa*, *iota*, and *lambda*) exhibited photoprotective properties in UVB-induced human keratinocytes (HaCaT) cells. Carrageenan significantly reduced the production of reactive oxygen species (ROS) and protected against the negative effects of UVB-induced apoptosis. Excess ROS buildup has been linked to various skin conditions, including cancer and aging of the skin. Antioxidants are typically thought of as protective measures against UV-related skin disorders as a result [143]. In HaCaT cells and mouse embryonic fibroblasts (MEFs), the antioxidative and photoprotective properties of a compound of κ -COSs and collagen peptide (CP) were shown. Through lowering the level of intracellular ROS, a compound of κ -COSs and CP (100 g/mL) may considerably attenuate UV-induced cell death and apoptosis in HaCaT and MEF. By inhibiting the mitogen-activated protein kinases (MAPKs) signaling pathway, a compound of κ -COSs and CP largely prevented the UV-induced decrease of type 1 pro-collagen and rise in MMP-1. A compound of CP and κ -COSs may have photoprotective properties against skin aging when used together [144].

### 4.3. Porphyran

Porphyran is a polisaccharide produced by *Porphyra* spp. The linear structure of the porphyran is composed of units of glycosidically substituted -D-galactopyranose at carbon 3 and -L-galactopyranose at carbon 4 in a repeated alternating disaccharide arrangement. Porphyran is a galactose that has undergone 6-*O*-sulfation of L-galactose units and 6-*O*-methylation of D-galactose units to a significant extent [3]. The main element of Porphyra is porphyran, a water-soluble sulfated polysaccharide found in the intercellular spaces and cell walls. Numerous studies have discovered various compositions and structures of porphyran obtained from various Porphyra species with interesting medicinal effects [145]. Porphyrans are used as a gelling agent, dietary supplement, antioxidant, and anti-allergic agent. They also exhibit tyrosinase inhibitory activity (the skin-whitening effects of red seaweed), protection from ultraviolet-B radiation, anti-inflammatory, antioxidant, and anticancer activity [5,134]. *Porphyra yezoensis* is used in cosmetic formulations by activating the NF-jB-dependent signal transduction pathway and downregulating the expression of inducible nitric oxide synthase (iNOS); it can decrease NO generation in LPS-induced RAW264.7 cells [146].

### 4.4. Galactan Sulfate

Complex galactans are also D/L-hybrids with galactose sugar substituted by other monosaccharides such as mannose, glucose, xylose and arabinose, as well as charged residues in the form of sulfate, methoxyl and pyruvic acid [147]. Galactans from red seaweeds are applied as gelling or thickening agents in cosmetic products [137,148]. 

**Table 5:** Polysaccharides from red seaweeds.

Species	Polysaccharides	Cosmetic Benefits	References
*Neopyropia yezoensis*	Porphyran	Anti-inflammatory, antioxidant, antiaging	[149]
*Halymenia durvillei*	Sulfated polysaccharides	Antiaging and antiwrinkle	[110]
*Gracilaria corticata*	Sulfated polysaccharides	Antioxidant	[115]
*Porphyra* *yezoensis*	Porphyran	Anti-inflammatory	[146]
*Eucheuma denticulatum*	Carrageenan	Antioxidant, photoprotection	[143]
*Acanthophora muscoides*	Sulfated polysaccharides Carrageenan	Anticoagulant, antinociceptive, anti-inflammatory, gel agents	[150]
*Chondrus crispus*	Carrageenan	Gel and thickening agent, skin moisturizer	[151]
*Gracilaria chouae*, *G. blodgetti*	Agar	Antioxidant, thickeners antitumor, radiation protector, antiaging	[137]
*Acanthophora muscoides*	Carrageenan	Anticoagulant, anti-inflammatory, gelling agents	[32]
*Pyropia yezoensis*	Porphyran	Antioxidant and anti-inflammatory	[32]
*Gigartina skottsbergii*	Galactan sulfate	Antioxidant	[152]

## 5. Prospects for Indonesia

**Figure 10:**
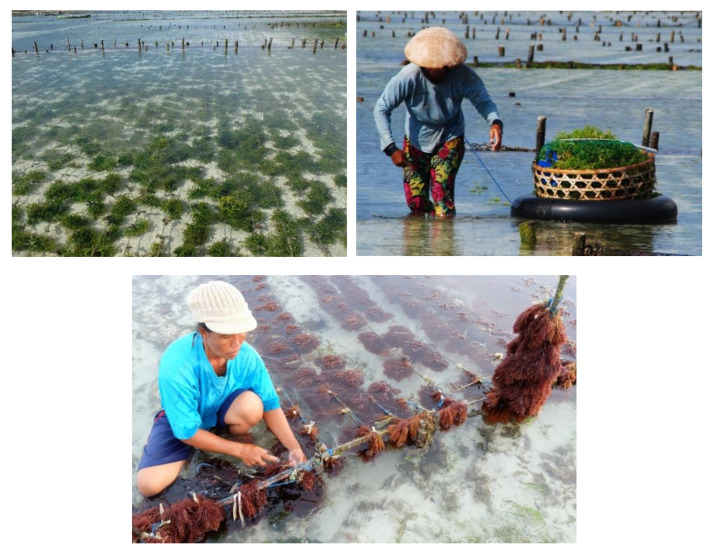
Activities of seaweed farming (*Eucheuma* spp. and *Halymenia* spp.) in Bali, Indonesia.

Indonesia is one of the megadiversity countries in world and the diversity of Indonesian marine life is bountiful. The Indonesian coastal zone is rich in tropical marine ecosystems such as estuarial beaches, mangroves, coral reefs, seagrass and seaweeds. The high level of biodiversity of the Indonesian fauna and flora provides opportunities for diversification of production. This provides opportunities for a wide range of species to be produced in aquaculture. The diversity of Indonesian seaweeds is abundant and red seaweeds are more diverse than green and brown seaweeds [120,121]. To date, only a few species of red seaweeds in Indonesia have been used for economic benefits as producers of phycocoloid compounds. Carragenophyte species *Kappaphycus alvarezii, K. striatus, Eucheuma denticulatum* and *E. cottonii* are cultured in coastal areas in 10 provinces in Indonesia [153]. *Gracilaria* spp. and *Gelidium* spp. are cultured in Indonesia for producing agar. Most *Gracilaria* culture is undertaken in brackish water ponds, usually in polyculture with milkfish, shrimp or other species [154]. Carragenan and agar are raw materials widely used in the food, pharmaceutical, cosmetic and biotechnology industries. Indonesia is one of the big five global producers of the red seaweeds *Kappaphycus* spp. and *Eucheuma* spp. and is a major producer of *Gracilaria* sp. worldwide [155]. Seaweeds aquaculture is a part of livelihood to increase household income in the coastal areas of Indonesia (Figure 10). 

Marine biodiversity provides promising sources of active compounds for cosmeceutical purposes. Among them, seaweeds represent a sustainable and renewable resource, gaining increasing attention for cosmeceutical applications. This review presents species of Indonesian red seaweeds’ potential as a producer of mycosporine-like amino acids and hydrogels. The general pattern of MAAs’ accumulation is a very variable and species-specific mechanism. These studies consider which species could potentially produce MAAs in a high concentration from different environments. There are a few steps for developing red seaweeds for cosmeceuticals in Indonesia. First, research on Rhodophyta is fundamental to expedite the opportunity to discover new chemical entities from Indonesia Rhodophyta. The results of this research will contribute to the selection of potential red seaweeds for further development. The existing potential will be utilized for the diversification of seaweed cultivation and development of industrial materials. Second, the introduction of potential seaweed cultivation farming through program socialization and seaweed farmer assistance is required. Third, the involvement of government and industry for application is needed.

Indonesia is a potential global and local market for cosmeceutical products. Revenue of the beauty and personal care market in Indonesia was about 7 billion USD in 2020. The Statista Consumer Market Outlook estimates that the number is predicted rise to roughly 9.6 billion USD by 2025 [156]. Skincare is one of the most profitable product categories, as it is projected to generate roughly 177 billion USD in 2025. Therefore, cosmeceuticals for photoprotectant and antiphotoaging effects and moisturizers are a potential market. Application of MAAs from red seaweed in cosmeceutical products is expected to increase due to their excellent properties as natural UV photoprotectors, such as high photo- and thermo-stability and the lack of oxidant photoproducts after UV absorption. In addition, in Muslim countries, most consumers will check products for the “halal” label, meaning that they comply with Islamic law and do not contain pork or derivates. Cosmeceuticals from seaweed meet the criteria of being halal, natural, safe, nontoxic and allergen-free. 

## 6. Conclusions

The interest in seaweeds for cosmeceuticals is increasing since lifestyle has become influenced by being “back to nature” and the demand for natural resources for health and life have increased. Cosmeceutical industries are exploring natural products and discovering new compounds derived from natural products due to consumers’ preference for natural cosmeceuticals. The advantages of seaweeds as natural resources are that they are rich in valuable cosmeceutical agents, versality, have many functions and sensory properties for skin care, have a wide distribution and are renewable resources. Seaweed provides natural compounds which can be used as ingredients, as additives, and as active agents in the formulation of skincare products. This review shows the importance of red seaweed-derived compounds in cosmeceuticals as photoprotectant agents, antiphotoaging agents and as source of hydrogels. Even though hydrocolloids from red seaweeds have already been used, mainly as thickening or gelling agents, they have a lot more unexplored potential, especially as sunscreens due to their photoprotectant and antiphotoaging effects. Natural compounds from seaweeds are effective and safe to be used in cosmeceuticals. Further studies are important to understand the mechanisms of actions of these compounds. In order to improve the quality of cosmetic products, more clinical trials have to be carried out to determine skin absorption, irritation, genetic and phototoxicity and allergen contents. Natural products have been proven characteristically safe and effective for various effects such as antiaging, antioxidant and UV-protective effects. They are abundant and sustainable in nature. The review clearly indicates that Rhodophyta are rich sources of MAAs with a UV-protective effect and antioxidant activity. Therefore, Rhodophyta is an excellent treasure trove for sunscreen and antiphotoaging applications. 

Indonesia is one of the big five producers of seaweeds worldwide. Research on the optimization of seaweed diversity for utilization as food, feed, pharmaceuticals and cosmeceuticals is important to maximize the potential of red seaweed in Indonesia for improving economic value. There are still large opportunities to explore MAAs from seaweed as antiphotoaging agents in the skincare and cosmetic industries. Furthermore, more studies need to be carried out on the sustainable culture of seaweeds and their optimization in order to obtain an optimal content of MAAs. 

## Figures and Tables

**Table 1:** Mycosporine-like Amino Acids (MAA) from red seaweed.

MAA	λmax (nm)	*m*/*z*
4-deoxygadusol	268	174.05
Gadusol	269	205.07
Mycosporine-taurine	309	295.07
Mycosporine-glycine	310	245.09
Mycosporine-alanine-glycine	310	259.11
Mycosporine-serine	310	275.12
Palythine	320	244.11
Bostrychines A	322	315.14
Bostrychines C	322	316.13
Prasiolin	324	333.11
Mycosporine-methylamine-threonine	328	304.16
Asterina-330	330	288.13
Palythinol	332	302.15
Aplysiapalythine A	332	289.14
Aplysiapalythine B	332	273.14
Bostrychines F	332	360.15
Bostrychines E	333	371.17
Mycosporine-2-glycine	334	306.14
Porphyra-334	334	364.14
Shinorine	334	332.12
Catenelline	334	382.1
Bostrychines B	335	417.17
Palythenic acid	337	332.16
Bostrychines D	337	418.16
Usujirene	357	284.14
Palythene	360	284.14

**Table 2:** Biogeography distribution of MAAs from *Bostrychia calliptera*.

Lineage	Geography	MAAs
1	North America, South America, Australia	Unpurified MAAs detected at 332 and 308 nm
2	Australia, Southeast Asia	Palythine-threonine
3	Central and South America	Palythine-threonine, Porphyra-334

**Table 3:** Antiphotoaging agents of various red seaweeds.

Species	Compound	Activity	References
*Gracilaria lemaneiformis*	Polysaccharides	Antiaging	[107]
*Porphyra yezoensis*	PYP1–5; Porphyra-334 Extract	Antiphotoaging Remediate skin aging	[87] [57]
*Porphyra umbilicalis*	Extract; polyphenols	Antioxidant, Antiaging properties	[71,108,109]
*Gelidium corneum*	Ethanol:_d_H_2_O (4:1) extract	Antioxidant	[71]
*Osmundea pinnatifida*	Ethanol:_d_H_2_O (4:1) extract	Antioxidant	[71]
*Porphyra tenera*	Porphyra-334; shinorine	Antiphotoaging	[93]
*Jania rubens*	Malonylshisonin; 4′′′-demalonylsalvianin	Antiaging	[97]
	Flavonoid	Antiwrinkle	[97]
*Halymenia durvillei*	Sulphate polysaccharides Methanol extract	Antiaging and antiwrinkle	[100,110,111]
*Corallina pilulifera*	Methanol extract	Antiaging	[112]
*Porphyra columbina*	Phenolic extracts	Photoprotectant, antiaging	[113]
*Gigartina skottsbergii*	Phenolic extracts	Photoprotectant, antiaging	[113,114]
*Sarcothalia radula*	Phenolic extracts	Photoprotectant, antiaging	[113]
*Gigartina chamisoi*	Extracts	Antioxidant, antiaging	[114]
*Gigartina radula*	Extracts	Antioxidant, antiaging	[114]
*Gigartina chilensis*	Extracts	Antioxidant, antiaging	[114]
*Gracilaria changii*	Polyphenol	Antioxidant	[101]
*Gracilaria corticata*	Sulfated polysaccharide (galactose)	Antioxidant	[115]
*Polysiphonia fucoides*	Phenolic from ethanolic extract	Antioxidant	[102]
*Furcellaria lumbricalis*	Aqueous extract	Antiaging	[116]
*Gracilaria birdiae*	Apigenin and gallic acid	Antioxidant	[117]
*Gracilaria cornea*	Apigenin and gallic acid	Antioxidant	[117]
*Gracilaria birdiae*	Sulfated polysaccharide	Antioxidant	[118]
*Pyrophia haitanesis*	Porphyrin	Antioxidant; antiaging	[119]
*Gelidium sesquipedale*	Extract	Restoring elasticity and softness	[109]
*Corallina officinalis*	Extract	Antioxidant; antiaging	[109]
*Palmaria palmata, Porhyra purpurea, Chondrus crispus, Mastocarpus stellatus, Gracilaria vermiculophylla*	Gallic acid, Protocatechuic acid, chlorogenic acids, gentisic acid	Antiaging	[102]

**Table 4:** Red seaweeds of Indonesia as potential MAAs producers.

Rhodophyta Collection	Type of MAA	References
*Acanthophora specifera*	AS, MG, PE, PL, PR, PI	[122]
*Ahnfeltiopsis* sp.	AS, PI, SH	[123]
*Amansia* sp.	AS, PR, PI, SH	[55]
*Amphiroa* sp.	PI, SH	[124]
*Dichotomaria marginata*	AS, PI, SH	[55]
*Eucheuma* sp.	PR	[125]
*Galaxaura* sp.	PR, SH	[122]
*Ganonema* sp.	PR, SH	[122]
*Gelidiella acerosa*	AS, PR, PI, SH	[122]
*Gelidium* sp.	AS, PL, PR, PI, SH	[126]
*Gracilaria* sp.	AS, MG, PE, PL, PR, PI, SH, US, PA	[62,127,128,129]
*Gymnogongrus* sp.	APA, APB, AS, M2G, MGV, MG, MMT, PE, PI, PGA, PS, PL, PR, SH, US	[130]
*Hypnea* sp.	AS, MG, PR, PI, SH	[131]
*Jania rubens*	AS, MG, PR, PI, SH	[55]
*Laurencia* sp.	AS, MG, PR, PI, SH, PL, US	[131,132]
*Mastocarpus stellatus*	APA, AS, MAG, PL, PR, PI, SH, US	[52,73,133]
*Palmaria palmata*	AS, MG, PE, PL, PR, PI, SH, US	[63,78]

AS: Asterina-330, APA: Aplysiapalythine A, APB: Aplysiapalythine B, M2G: Mycosporine-2-glycine, MGV: Mycosporine-glycine-valine, MAG: Mycosporine-alanine-glycine, MG: Mycosporine-glycine, MMT: Mycosporine-methylamine threonine, PE: Palythene, PA: Palythenic acid, PI: Palythine, PGA: Palythine-glutamic acid, PS: Palythine-serine, PL: Palytinol, PR: Porphyra-334, SH: Shinorine, US: Usujirene.

## Data Availability

Not applicable.

## References

[1] FDA. https://www.fda.gov/cosmetics.

[2] Thomas N.V., Kim S.-K. (2013). Beneficial effects of marine algal compounds in cosmeceuticals. Mar. Drugs.

[3] Cotas J., Leandro A., Pacheco D., Gonçalves A.M.M., Pereira L. (2020). A comprehensive review of the nutraceutical and therapeutic applications of red seaweeds (Rhodophyta). Life.

[4] Thiyagarasaiyar K., Goh B.-H., Jeon Y.-J., Yow Y.-Y. (2020). Algae metabolites in cosmeceutical: An overview of current applications and challenges. Mar. Drugs.

[5] López-Hortas L., Flórez-Fernández N., Torres M.D., Ferreira-Anta T., Casas M.P., Balboa E.M., Falqué E., Domínguez H. (2021). Applying seaweed compounds in cosmetics, cosmeceuticals and nutricosmetics. Mar. Drugs.

[6] GlobeNewswire. https://www.globenewswire.com/news-release/2021/10/05/2308851/0/en/Cosmetics-Market-Size-to-Worth-Around-US-480-4-Bn-by-2030.html.

[7] Grand View Research. https://www.grandviewresearch.com/industry-analysis/skin-care-products-market.

[8] Ahmed I.A., Mikail M.A., Zamakshshari N., Abdullah A.H. (2020). Natural anti-aging skincare: Role and potential. Biogerontology.

[9] Pimentel F.B., Alves R.C., Rodrigues F., Oliveira M.B.P.P. (2018). Macroalgae-derived ingredients for cosmetic industry—An update. Cosmetics.

[10] Lourenço-Lopes C., Fraga-Corral M., Jimenez-Lopez C., Pereira A., Garcia-Oliveira P., Carpena M., Prieto M., Simal-Gandara J. (2020). Metabolites from macroalgae and its applications in the cosmetic industry: A circular economy approach. Resources.

[11] Demont A. (2020). The science behind the ingredients. Cosmet. Lab..

[12] Thompson C.C., Kruger R.H., Thompson F.L. (2017). Unlocking marine biotechnology in the developing world. Trends Biotechnol..

[13] Pallela R., Na-Young Y., Kim S. (2010). Anti-photoaging and photoprotective compounds derived from marine organisms. Mar. Drugs.

[14] Guillerme J.-B., Couteau C., Coiffard L. (2017). Applications for marine resources in cosmetics. Cosmetics.

[15] Brunt E.G., Burgess J.G. (2018). The promise of marine molecules as cosmetic active ingredients. Int. J. Cosmet. Sci..

[16] Alves A., Sousa E., Kijjoa A., Pinto M. (2020). Marine-derived compounds with potential use as cosmeceuticals and nutricosmetics. Molecules.

[17] Sotelo C.G., Blanco M., Ramos P., Vázquez J.A., Perez-Martin R.I. (2021). Sustainable sources from aquatic organisms for cosmeceuticals ingredients. Cosmetics.

[18] Morais T., Cotas J., Pacheco D., Pereira L. (2021). Seaweeds compounds: An eco-sustainable source of cosmetic ingredients?. Cosmetics.

[19] Chan C.-X., Ho C.-L., Phang S.-M. (2006). Trends in seaweed research. Trends. Plant Sci..

[20] Sathianeson S., Siddik A.A., Baakdah M., Al-Sofyani A.A., Nabti E. (2017). An introduction to the ecological significance of seaweeds on coastal ecosystems. Biotechnological Applications of Seaweeds.

[21] Sudatti D.B., Duarte H.M., Soares A.R., Salgado L.T., Pereira R.C. (2020). New ecological role of seaweed secondary metabolites as auto toxic and allelopathic. Front. Plant Sci..

[22] Bhatia S., Garg A., Sharma K., Kumar S., Sharma A., Purohit A.P. (2011). Mycosporine and mycosporine-like amino acids: A paramount tool against ultra violet irradiation. Pharmacogn. Rev..

[23] Geraldes V., Pinto E. (2021). Mycosporine-like amino acids (MAAs): Biology, chemistry and identification features. Pharmaceuticals.

[24] Baweja P., Kumar S., Sahoo D., Levine I., Fleurence F., Levine I. (2016). Biology of seaweed. Seaweed in Health and Disease Prevention.

[25] Pérez-Lloréns J.L., Mouritsen O.G., Rhatigan P., Cornish M.L., Critchley A.T. (2020). Seaweeds in mythology, folklore, poetry, and life. J. Appl. Phycol..

[26] El-Beltagi H.S., Mohamed A.A., Mohamed H.I., Ramadan K.M.A., Barqawi A.A., Mansour A.T. (2022). Phytochemical and potential properties of seaweeds and their recent applications: A review. Mar. Drugs.

[27] Rebours C., Marinho-Soriano E., Zertuche-González J.A., Hayashi L., Vásquez J.A., Kradolfer P., Soriano G., Ugarte R., Abreu M.H., Bay-Larsen I. (2014). Seaweeds: An opportunity for wealth and sustainable livelihood for coastal communities. J. Appl. Phycol..

[28] Chung I.K., Sondak C.F., Beardall J. (2017). The future of seaweed aquaculture in a rapidly changing world. Eur. J. Phycol..

[29] Kumari P., Kumar M., Gupta V., Reddy C.R.K., Jha B. (2010). Tropical marine macroalgae as potential sources of nutritionally important PUFAs. Food Chem..

[30] Pereira L. (2016). Edible Seaweed of the World.

[31] Kim S.K. (2014). Marine cosmeceuticals. J. Cosmet. Dermatol..

[32] Pereira L. (2018). Seaweeds as source of bioactive substances and skin care therapy—Cosmeceuticals, algotheraphy, and thalassotherapy. Cosmetics.

[33] Kalasariya H.S., Yadav V.K., Yadav K.K., Tirth V., Algahtani A., Islam S., Gupta N., Jeon B.H. (2021). Seaweed-based molecules and their potential biological activities: An eco-sustainable cosmetic. Molecules.

[34] Wang H.-M.D., Chen C.-C., Huynh P., Chang J.-S. (2015). Exploring the potential of using algae in cosmetics. Bioresour. Technol..

[35] Druehl L.D., Clarkston B.E. (2016). Pacific Seaweeds: A Guide to Common Seaweeds of the West Coast.

[36] Bunker F., Brodie J.A., Maggs C.A., Bunker A.R. (2020). Seaweeds of Britain and Ireland.

[37] Jha B., Reddy C., Thakur M.C., Rao M.U. (2009). Seaweeds of India: The Diversity and Distribution of Seaweeds of Gujarat Coast.

[38] Mouritsen O. (2013). Marine macroalgae benefit people culturally, industry, nutritionally, and ecologically. Am. Sci..

[39] Gurgel C.F.D., Lopez-Bautista J. (2007). Red algae. Encyclopedia of Life Science.

[40] Carpena M., Garcia-Perez P., Garcia-Oliviera P., Chamorro F., Otero P., Lourenco-Lopes C., Cao H., Simal-Gandara J., Prieto M.A. (2022). Biological properties and potential of compounds extracted from red seaweeds. Phytochem. Rev..

[41] Torres M.D., Flórez-Fernández N., Domínguez H. (2019). Integral utilization of red seaweed for bioactive production. Mar. Drugs.

[42] Shannon E., Abu-Ghannam N. (2019). Seaweeds as nutraceuticals for health and nutrition. Phycologia.

[43] Salehi B., Sharifi-Rad J., Seca A.M.L., Pinto D.C.G.A., Michalak I., Trincone A., Mishra A.P., Nigam M., Zam W., Martins N. (2019). Current trends on seaweeds: Looking at chemical composition, phytopharmacology, and cosmetic applications. Molecules.

[44] Pangestuti R., Siahaan E.A., Kim S.K. (2018). Photoprotective substances derived from marine algae. Mar. Drugs.

[45] Barnes P.W., Williamson C.E., Lucas R.M., Robinson S.A., Madronich S., Paul N.D., Bornman J.F., Bais A.F., Sulzberger B., Wilson S.R. (2019). Ozone depletion, ultraviolet radiation, climate change and prospects for a sustainable future. Nat. Sustain..

[46] Núñez-Pons L., Avila C., Romano G., Verde C., Giordano D. (2018). UV-protective compounds in marine organisms from the Southern Ocean. Mar. Drugs.

[47] Álvarez-Gómez F., Korbee N., Casas-Arrojo V., Abdala-Díaz R.T., Figueroa F.L. (2019). UV photoprotection, cytotoxicity and immunology capacity of red algae extracts. Molecules.

[48] Bonomi-Barufi J., Figueroa F.L., Korbee N., Momoli M.M., Martins A.P., Colepicolo P., van Sluys M.A., Oliveira M.C. (2020). How macroalgae can deal with radiation variability and photoacclimation capacity: The example of *Gracilaria tenuistipitata* (Rhodophyta) in laboratory. Algal Res..

[49] Vladkova T., Georgieva N., Staneva A., Gospodinova D. (2022). Recent progress in antioxidant active substances from marine biota. Antioxidants.

[50] Lalegerie F., Lajili S., Bedoux G., Taupin L., Stiger-Pouvreau V., Connan S. (2019). Photo-protective compounds in red macroalgae from Brittany: Considerable diversity in mycosporine-like amino acids (MAAs). Mar. Environ. Res..

[51] Guihéneuf F., Gietl A., Stengel D.B. (2018). Temporal and spatial variability of mycosporine-like amino acids and pigments in three edible red seaweeds from western Ireland. J. Appl. Phycol..

[52] Orfanoudaki M., Hartmann A., Karsten U., Ganzera M. (2019). Chemical profiling of mycosporine-like amino acids in twenty-three red algal species. J. Phycol..

[53] Geraldes V., de Medeiros L.S., Jacinavicius F.R., Long P.F., Pinto E. (2020). Development and validation of a rapid LC-MS/MS method for the quantification of mycosporines and mycosporine-like amino acids (MAAs) from cyanobacteria. Algal Res..

[54] Kim B.K., Park M.O., Min J.O., Kang S.H., Shin K.H., Yang E.J., Ha S.Y. (2022). The interplay of mycosporine-like amino acids between phytoplankton groups and northern krill (*Thysanoessa* sp.) in a high-latitude Fjord (Kongsfjorden, Svalbard). Mar. Drugs.

[55] Sun Y., Zhang N., Zhou J., Dong S., Zhang X., Guo L., Guo G. (2020). Distribution, contents, and types of mycosporine-like amino acids (MAAs) in marine macroalgae and a database for MAAs based on these characteristics. Mar. Drugs.

[56] Sánchez-Lamar Á., González-Pumariega M., Fuentes-León F., Vernhes Tamayo M., Schuch A.P., Menck C.F. (2017). Evaluation of genotoxic and DNA photo-protective activity of *Bryothamnion triquetrum* and *Halimeda incrassata* seaweeds extracts. Cosmetics.

[57] Park J., Lee H., Choi S., Pandey L.K., Depuydt S., De Saeger J., Park J.T., Han T. (2021). Extracts of red seaweed, *Pyropia yezoensis*, inhibit melanogenesis but stimulate collagen synthesis. J. Appl. Phycol..

[58] David S.R., Baharulnizam N.B., Rajabalaya R. (2022). A review on biological assays of red algae marine compounds: An insight into skin whitening activities. J. Herb. Med..

[59] La Barre S., Roullier C., Boustie J. (2014). Mycosporine-like amino acids (MAAs) in biological photosystems. Outstanding Marine Molecules.

[60] Bedoux G., Pliego-Cortés H., Dufau C., Hardouin K., Boulho R., Freile-Pelegrin Y., Robledo D., Bourgougnon N., Bourgougnon N. (2020). Production and properties of mycosporine-like amino acids isolated from seaweeds. Advances in Botanical Research.

[61] Cardozo K.H., Marques L.G., Carvalho V.M., Carignan M.O., Pinto E., Marinho-Soriano E., Colepicolo P. (2011). Analyses of photoprotective compounds in red algae from the Brazilian coast. Rev. Bras. Farmacogn..

[62] Sun Y., Han X., Hu Z., Cheng T., Tang Q., Wang H., Deng X., Han X. (2021). Extraction, isolation and characterization of mycosporine-like amino acids from four species of red macroalgae. Mar. Drugs.

[63] Nishida Y., Kumagai Y., Michiba S., Yasui H., Kishimura H. (2020). Efficient extraction and antioxidant capacity of mycosporine-like amino acids from red alga Dulse *Palmaria palmata* in Japan. Mar. Drugs.

[64] Hartmann A., Becker K., Karsten U., Remias D., Ganzera M. (2015). Analysis of mycosporine-like amino acids in selected algae and cyanobacteria by hydrophilic interaction liquid chromatography and a novel MAAs from the red alga *Catenella repens*. Mar. Drugs.

[65] Orfanoudaki M., Hartmann A., Miladinovic H., Nguyen N.H., Karsten U., Ganzera M. (2019). Bostrychines A–F, six novel mycosporine-like amino-acids and a novel betaine from the red alga *Bostrychia scorpioides*. Mar. Drugs.

[66] Navarro N.P., Figueroa F.L., Korbee N., Bonomi J., Álvarez-Gómez F., de la Coba F., Rastogi R.P. (2018). Mycosporine-like amino acids from red algae to develop natural UV sunscreens. Sunscreens: Source, Formulation, Efficacy and Recommendations. Biochemistry Research Trends.

[67] Pliego-Cortés H., Bedoux G., Boulho R., Taupin L., Freile-Pelegrín Y., Bourgougnon N., Robledo D. (2019). Stress tolerance and photoadaptation to solar radiation in *Rhodymenia pseudopalmata* (Rhodophyta) through mycosporine-like amino acids, phenolic compounds, and pigments in an integrated multi-trophic aquaculture system. Algal Res..

[68] Schmid M., Stengel D.B. (2015). Intra-thallus differentiation of fatty acid and pigment profiles in some temperate Fucales and Laminariales. J. Phycol..

[69] Diehl N., Michalik D., Zuccarello G.C., Karsten U. (2019). Stress metabolite pattern in the eulittoral red alga *Pyropia plicata* (Bangiales) in New Zealand–mycosporine-like amino acids and heterosides. J. Exp. Mar. Biol. Ecol..

[70] Véliz K., Chandía N., Karsten U., Lara C., Thiel M. (2019). Geographic variation in biochemical and physiological traits of the red seaweeds *Chondracanthus chamissoi* and *Gelidium lingulatum* from the south east Pacific coast. J. Appl. Phycol..

[71] Vega J., Bonomi-Barufi J., Gómez-Pinchetti J.L., Figueroa F.L. (2020). Cyanobacteria and red macroalgae as potential sources of antioxidants and UV radiation-absorbing compounds for cosmeceutical applications. Mar. Drugs.

[72] Schneider G., Figueroa F.L., Vega J., Chaves P., Álvarez-Gómez F., Korbee N., Bonomi-Barufi J. (2020). Photoprotection properties of marine photosynthetic organisms grown in high ultraviolet exposure areas: Cosmeceutical applications. Algal Res..

[73] Zwerger M., Ganzera M. (2022). Fast and efficient separation of eleven mycosporine-like amino acids by UHPLC-DAD and their quantification in diverse red algae. Mar. Drugs.

[74] Orfanoudaki M., Hartmann A., Kamiya M., West J., Ganzera M. (2020). Chemotaxonomic study of *Bostrychia* spp. (Ceramiales, Rhodophyta) based on their mycosporine-like amino acid content. Molecules.

[75] Jofre J., Celis-Plá P.S., Figueroa F.L., Navarro N.P. (2020). Seasonal variation of mycosporine-like amino acids in three subantarctic red seaweeds. Mar. Drugs.

[76] Figueroa F.L., Domínguez-González B., Korbee N. (2014). Vulnerability and acclimation to increased UVB radiation in three intertidal macroalgae of different morpho-functional groups. Mar. Environm. Res..

[77] Gambichler V., Zuccarello G.C., Karsten U. (2021). Seasonal changes in stress metabolites of native and introduced red algae in New Zealand. J. Appl. Phycol..

[78] Lalegerie F., Stiger-Pouvreau V., Connan S. (2020). Temporal variation in pigment and mycosporine-like amino acid composition of the red macroalga *Palmaria palmata* from Brittany (France): Hypothesis on the MAA biosynthesis pathway under high irradiance. J. Appl. Phycol..

[79] Barufi J.B., Mata M.T., Oliveira M.C., Figueroa F.L. (2012). Nitrate reduces the negative effect of UV radiation on photosynthesis and pigmentation in *Gracilaria tenuistipitata* (Rhodophyta): The photoprotection role of mycosporine-like amino acids. Phycologia.

[80] Barceló-Villalobos M., Figueroa F.L., Korbee N., Álvarez-Gómez F., Abreu M.H. (2017). Production of mycosporine-like amino acids from *Gracilaria vermiculophylla* (Rhodophyta) cultured through one year in an integrated multi-trophic aquaculture (IMTA) system. Mar. Biotech..

[81] Navarro N.P., Korbee N., Jofre J., Figueroa F.L. (2021). Short-term variations of mycosporine-like amino acids in the carrageenan-producing red macroalga *Mazzaella laminarioides* (Gigartinales, Rhodophyta) are related to nitrate availability. J. Appl. Phycol..

[82] Oren A., Gunde-Cimerman N. (2007). Mycosporines and mycosporine-like amino acids: UV protectants or multipurpose secondary metabolites?. FEMS Microbiol. Lett..

[83] Rosic N.N. (2019). Mycosporine-like amino acids: Making the foundation for organic personalised sunscreens. Mar. Drugs.

[84] Suh S.-S., Hwang J., Park M., Seo H.H., Kim H.-S., Lee J.H., Moh S.H., Lee T.-K. (2014). Anti-inflammation activities of mycosporine like amino acids (MAAs) in response to UV radiation suggest potential anti-skin aging activity. Mar. Drugs.

[85] Kim S.Y., Cho W.K., Kim H.-I., Paek S.H., Jang S.J., Jo Y., Choi H., Lee J.H., Moh S.H. (2021). Transcriptome profiling of human follicle dermal papilla cells in response to porphyra-334 treatment by RNA-seq. Evid. Based Complement. Altern. Med..

[86] Vega J., Schneider G., Moreira B.R., Herrera C., Bonomi-Barufi J., Figueroa F.L. (2021). Mycosporine-like amino acids from red macroalgae: UV-photoprotectors with potential cosmeceutical applications. Appl. Sci..

[87] Ryu J., Park S.-J., Kim I.-H., Choi Y.H., Nam T.-J. (2014). Protective effect of prophyra-334 on UVA-induced photoaging in human skin fibroblast. Int. J. Mol. Med..

[88] Orfanoudaki M., Hartmann A., Alilou M., Gelbrich T., Planchenault P., Derbré S., Schinkovitz A., Richomme P., Hensel A., Ganzera M. (2019). Absolute configuration of mycosporine-like amino acids, their wound healing properties and in vitro anti-aging effects. Mar. Drugs.

[89] Lawrence K., Gacesa R., Long P., Young A. (2018). Molecular photoprotection of human keratinocytes in vitro by the naturally occurring mycosporine-like amino acid palythine. Br. J. Dermatol..

[90] Rosic N.N. (2021). Recent advances in the discovery of novel marine natural products and mycosporine-like amino acid UV-absorbing compounds. Appl. Microbiol. Biotechnol..

[91] Gunn D.A., Christensen K., Farage M.A., Miller K.W., Maibach H.I. (2017). Skin aging and health. Textbook of Aging Skin.

[92] Kageyama H., Waditee-Sirisattha R. (2019). Antioxidative, anti-inflammatory, and anti-aging properties of mycosporine-like amino acids: Molecular and cellular mechanisms in the protection of skin-aging. Mar. Drugs.

[93] Rui Y., Zhaohui Z., Wenshen S., Bafang L., Hu H. (2019). Protective effect of MAAs extracted from *Porphyra tenera* against UV irradiation-induced photoaging in mouse skin. J. Photochem. Photobiol. B Biol..

[94] Balboa E.M., Conde E., Soto M.L., Perez-Armada L., Dominguez H., Kim S.K. (2015). Cosmetics from marine sources. Handbook of Marine Biotechnology.

[95] Resende D.I.S.P., Ferreira M., Magalhaes C., Lobo J.M.S., Sousa E., Almeida I.F. (2021). Trends in the use of marine ingredients in anti-aging cosmetics. Algal Res..

[96] Janarthanan M., Kumar M.S. (2019). Ontogenesis of textile face mask using cotton fabric by treating with red seaweed extract for cosmetotextile applications. J. Text. Inst..

[97] Dixit D., Reddy C.R.K. (2017). Non-targeted secondary metabolite profile study for deciphering the cosmeceutical potential of red marine macro alga *Jania rubens*—An LCMS-based approach. Cosmetics.

[98] Pangestuti R., Shin K.-H., Kim S.-K. (2021). Anti-photoaging and potential skin health benefits of seaweeds. Mar. Drugs.

[99] Pangestuti R., Kim S.-K., Kim S.-K. (2014). Biological activities of carrageenan. Advances in Food and Nutrition Research.

[100] Matanjun P., Mohamed S., Mustapha N.M., Muhammad K., Ming C.K. (2008). Antioxidant activities and phenolics content of eight species of seaweeds from north Borneo. J. Appl. Phycol..

[101] Chan P.T., Matanjun P., Yasir S.M., Tan T.S. (2015). Antioxidant activities and polyphenolics of various solvent extracts of red seaweed, *Gracilaria changii*. J. Appl. Phycol..

[102] Farvin K.H.S., Jacobsen C. (2013). Phenolic compounds and antioxidant activities of selected species of seaweeds from Danish coast. Food Chem..

[103] Hwang E., Park S.Y., Lee H.J., Lee T.Y., Sun Z.W., Yi T.H. (2014). Gallic acid regulates skin photoaging in UVB-exposed fibroblast and hairless mice. Phytother. Res..

[104] Shin S., Cho S.H., Park D., Jung E. (2020). Anti-skin aging properties of protocatechuic acid in vitro and in vivo. J. Cosmet. Dermatol..

[105] Girsang E., Ginting C.N., Lister I., Gunawan K.Y., Widowati W. (2021). Anti-inflammatory and antiaging properties of chlorogenic acid on UV-induced fibroblast cell. PeerJ.

[106] Michalun M.V., Dinardo J.C. (2014). Skin Care and Cosmetic Ingredients Dictionary.

[107] Wang X., Zhang Z., Zhou H., Sun X., Chen X., Xu N. (2019). The anti-aging effects of *Gracilaria lemaneiformis* polysaccharide in *Caenorhabditis elegans*. Int. J. Biol. Macromol..

[108] Mercurio D.G., Wagemaker T.A.L., Alves V.M., Benevenuto C.G., Gaspar L.R., Maia Campos P.M.B.G. (2015). In vivo photoprotective effects of cosmetic formulations containing UV filters, vitamins, *Ginkgo biloba* and red algae extracts. J. Photochem. Photobiol. B Biol..

[109] La-Mer. https://www.la-mer.com/en/about-la-mer/algae-seaweed-and-more/.

[110] SpecialChem—The Material Selection Platform: Cosmetics Ingredients. ExpoZen—GreenTech (specialchem.com). https://cosmetics.specialchem.com/product/i-greentech-expozen.

[111] ExpoZen. https://asharrison.com.au/expozen-gb/.

[112] Ryu B., Qian Z., Kim M., Nam K.W., Kim S. (2009). Anti-photoaging activity and inhibition of matrix metalloproteinase (MMP) by marine red alga, *Corallina pilulifera* methanol extract. Radiat. Phys. Chem..

[113] Guinea M., Franco V., Araujo-Bazan L., Rodriguez-Martin I., Gonzales S. (2012). In vivo UVB-photoprotective activity of extracts from commercial marine macroalgae. Food Chem. Toxicol..

[114] Ortiz-Viedma J., Aguilera J.M., Flores M., Lemus-Mondaca R., Larrazabal M.J., Miranda J.M., Aubourg S.P. (2021). Protective effect of red algae (*Rhodophyta*) extracts on essential dietary components of heat-treated Salmon. Antioxidants.

[115] Seedevi P., Moovendhan M., Viramani S., Shanmugam A. (2017). Bioactive potential and structural chracterization of sulfated polysaccharide from seaweed (*Gracilaria corticata*). Carbohydr. Polym..

[116] Al-Bader T., Byrne A., Gillbro J., Mitarotonda A., Metois A., Vial F., Rawlings A.V., Laloeuf A. (2012). Effect of cosmetic ingredients as anticellulite agents: Synergistic action of actives with in vitro and *in vivo* efficacy. J. Cosmet. Dermatol..

[117] Souza B.W.S., Cerqueira M.A., Martins J.T., Quintas M.A.C., Ferreira A.C.S., Teixeira J.A., Vicente A.A. (2011). Antioxidant potential of two red seaweeds from the Brazilian Coasts. J. Agric. Food Chem..

[118] Souza B.W.S., Cerqueira M.A., Bourbon A.I., Pinheiro A.C., Martins J.T., Teixeira J.A., Coimbra M.A., Vicente A.A. (2012). Chemical characterization and antioxidant activity of sulfated polysaccharide from the red seaweed *Gracilaria birdiae*. Food Hydrocoll..

[119] Zhang Z., Wang X., Pan Y., Wang G., Mao G. (2020). The degraded polysaccharide from *Pyropia haitanensis* represses amyloid beta peptide-induced neurotoxicity and memory in vivo. Int. J. Biol. Macromol..

[120] Kasanah N., Setyadi, Triyanto, Trialfhianty T.I. (2018). Indonesian Seaweeds: Biodiversity of Seaweeds in Gunung Kidul, Yogyakarta.

[121] Kasanah N., Ulfah M., Nugroho A., Wijnana A.P.A., dan Triyanto (2020). Indonesian Seaweeds: Biodiversity of Seaweeds in East Nusa Tenggara.

[122] Karsten U., Sawall T., Wiencke C. (2006). A survey of the distribution of UV-absorbing substances in tropical macroalgae. Phycol. Res..

[123] De la Coba F., Aguilera J., Figueroa F.L., De Gálvez M.V., Herrera E. (2009). Antioxidant activity of mycosporine-like amino acids isolated from three red macroalgae and one marine lichen. J. Appl. Phycol..

[124] Gröniger A., Sinha R.P., Klisch M., Häder D.P. (2000). Photoprotective compounds in cyanobacteria, phytoplankton and macroalgae—A database. J. Photochem. Photobiol. B Biol..

[125] Niu M.Y., Zhang Z.H., Gao M., Bu L., Zhang M.M. (2014). Optimization the extraction process of mycosporine-like amino acids from *Eucheuma*. Acad. Period. Farm Prod. Process..

[126] Quintano E., Celis-PláP S.M., Martínez B., Díez I., Muguerza N., Figueroa F.L., Gorostiaga J.M. (2019). Ecophysiological responses of a threatened red alga to increased irradiance in an in situ transplant experiment. Mar. Environ. Res..

[127] Figueroa F.L., Israel A., Neori A., Martínez B., Malta E.J., Put A., Inken S., Marquardt R., Abdala-Díaz R., Korbee N. (2010). Effect of nutrient supply on photosynthesis and pigmentation to short-term stress (UV radiation) in *Gracilaria conferta* (Rhodophyta). Mar. Pollut. Bull..

[128] Jin N.N., Zhang Z.H., Li B.F. (2012). The constitutes and extraction analysis of mycosporine-like amino acids (MAAs) in the Gracilariaceae. Mar. Sci..

[129] Torres P.B., Chow F., Ferreira M.J.P., dos Santos D.Y.A.C. (2016). Mycosporine-like amino acids from *Gracilariopsis tenuifrons* (Gracilariales, Rhodophyta) and its variation under high light. J. Appl. Phycol..

[130] Parailloux M., Godin S., Fernandes S.C., Lobinski R. (2020). Untargeted analysis for mycosporines and mycosporine-like amino acids by hydrophilic interaction liquid chromatography (HILIC)—Electrospray orbitrap MS2/MS3. Antioxidants.

[131] Briani B., Sissini M.N., Lucena L.A., Batista M.B., Costa I.O., Nunes J.M., Schmitz C., Ramlov F., Maraschin M., Korbee N. (2018). The influence of environmental features in the content of mycosporine-like amino acids in red marine algae along the Brazilian coast. J. Phycol..

[132] Stein E.M., Marques L.G., Fujii M.T., Colepicolo P. (2013). Mycosporine-like amino acids (MAAs): Qualitative analysis of photoprotective compounds in *Laurencia* complex seaweed species (Ceramiales, Rhodophyta) from Espírito Santo State, Brazil. Planta Med..

[133] Athukorala Y., Trang S., Kwok C., Yuan Y.V. (2016). Antiproliferative and antioxidant activities and mycosporine-like amino acid profiles of wild-harvested and cultivated edible Canadian marine red macroalgae. Molecules.

[134] Kim J.H., Lee J.E., Kim K.H., Kang N.J. (2018). Beneficial effects of marine algae-derived carbohydrates for skin health. Mar. Drugs.

[135] Xie X.T., Zhang X., Liu Y., Chen X.Q., Cheong K.L. (2020). Quantification of 3, 6-anhydro-galactose in red seaweed polysaccharides and their potential skin-whitening activity. 3 Biotech.

[136] Wang L., Lee W., Oh J.Y., Cui Y.R., Ryu B., Jeon Y.J. (2018). Protective effect of sulfated polysaccharides from celluclast-assisted extract of *Hizikia fusiforme* against ultraviolet B-Induced skin damage by regulating NF-κB, AP-1, and MAPKs signaling pathways in vitro in human dermal fibroblasts. Mar. Drugs.

[137] Fernando I.P.S., Kim K.N., Kim D., Jeon Y.J. (2018). Algal polysaccharides: Potential bioactive substances for cosmeceutical applications. Crit. Rev. Biotechnol..

[138] Van de Velde F., Knutsen S.H., Usov A.I., Rollema H.S., Cerezo A.S. (2002). H-1 and C-13 high resolution NMR spectroscopy of carrageenans: Application in research and industry. Trends Food Sci. Technol..

[139] Kasanah N., Seto D.S., Khotimah H., Triyanto (2022). Edible Seaweed: Chemistry, Bioactivity, and Toxicity.

[140] Langford A., Zhang J., Waldron S., Julianto B., Siradjuddin I., Neish I., Nuryartono N. (2022). Price analysis of the Indonesian carrageenan seaweed industry. Aquaculture.

[141] Ahmed A.B.A., Adel M., Karimi P., Peidayesh M., Kim S.-K. (2014). Pharmaceutical, cosmeceutical, and traditional applications of marine carbohydrates. Advances in Food and Nutrition Research.

[142] Shafie M.H., Kamal M.L., Zulkiflee F.F., Hasan S., Uyop N.H., Abdullah S., Zafarina Z. (2022). Application of Carrageenan extract from red seaweed (Rhodophyta) in cosmetic products: A review. J. Indian Chem. Soc..

[143] Thevanayagam H., Mohamed S.M., Chu W.L. (2014). Assessment of UVB-photoprotective and antioxidative activities of carrageenan in keratinocytes. J. Appl. Phycol..

[144] Ren S.W., Li J., Wang W., Guan H.S. (2010). Protective effects of kappa-ca3000+CP against ultraviolet-induced damage in HaCaT and MEF cells. J. Photochem. Photobiol. B.

[145] Qiu Y., Jiang H., Fu L., Ci F., Mao X. (2021). Porphyran and oligo-porphyran originating from red algae Porphyra: Preparation, biological activities, and potential applications. Food Chem..

[146] Jiang Z., Hama Y., Yamaguchi K., Oda T. (2012). Inhibitory effect of sulphated polysaccharide porphyran on nitric oxide production in lipopolysaccharide-stimulated RAW264. 7 macrophages. J. Biochem..

[147] Lim Y.Y., Lee W.K., Leow A.T.C., Namasivayam P., Abdullah J.O., Ho C.L. (2018). Sulfated galactans from red seaweeds and their potential applications. PJSRR.

[148] Delattre C., Fenoradosoa T.A., Michaud P. (2011). Galactans: An overview of their most important sourcing and applications as natural polysaccharides. Braz. Arch. Biol. Technol..

[149] Isaka S., Cho K., Nakazono S., Abu R., Ueno M., Kim D., Oda T. (2015). Antioxidant and anti-inflammatory activities of porphyran isolated from discolored nori (*Porphyra yezoensis*). Int. J. Biol. Macromol..

[150] Rodrigues J.A.G., de Queiroz I.N.L., Quinderé A.L.G., Benevides N.M.B., Tovar A.M.F., de Souza Mourão P.A. (2016). Extraction and structural properties of *Acanthophora muscoides* (Rhodophyceae) extracellular matrix sulfated polysaccharides and their effects on coagulation. Acta Sci. Technol..

[151] Stengel D.B., Connan S. (2015). Marine algae: A source of biomass for biotechnological applications. Natural Products from Marine Algae—Methods in Molecular Biology.

[152] Barahona T., Encinas M.V., Mansilla A., Matsuhiro B., Zúñiga E.A. (2012). A sulfated galactan with antioxidant capacity from the green variant of tetrasporic *Gigartina skottsbergii* (Gigartinales, Rhodophyta). Carbohydrate Res..

[153] Waters T.J., Lionata H., Prasetyo Wibowo T., Jones R., Theuerkauf S., Usman S., Amin I., Ilman M. (2019). Coastal Conservation and Sustainable Livelihoods through Seaweed Aquaculture in Indonesia: A Guide for Buyers, Conservation Practitioners, and Farmers.

[154] Rimmer M.A., Sugama K., Rakhmawati D., Rofiq R., Habgood R.H. (2013). A review and SWOT analysis of aquaculture development in Indonesia. Rev. Aquac..

[155] Rimmer M.A., Larson S., Lapong I., Purnomo A.H., Pong-Masak P.R., Swanepoel L., Paul N.A. (2021). Seaweed aquaculture in Indonesia contributes to social and economic aspects of livelihoods and community wellbeing. Sustainability.

[156] Statista. https://www.statista.com/statistics/585522/global-value-cosmetics-market/.

